# Propolis-Based Nanofiber Patches to Repair Corneal Microbial Keratitis

**DOI:** 10.3390/molecules26092577

**Published:** 2021-04-28

**Authors:** Songul Ulag, Elif Ilhan, Ramazan Demirhan, Ali Sahin, Betul Karademir Yilmaz, Burak Aksu, Mustafa Sengor, Denisa Ficai, Aurel Mihail Titu, Anton Ficai, Oguzhan Gunduz

**Affiliations:** 1 Center for Nanotechnology & Biomaterials Application and Research (NBUAM), Marmara University, 34722 Istanbul, Turkey; ulagitu1773@gmail.com (S.U.); eliffguven@gmail.com (E.I.); r.demirhan@outlook.com (R.D.); mustafasengor@gmail.com (M.S.); 2 Metallurgical and Materials Engineering, Institute of Pure and Applied Sciences, Marmara University, 34722 Istanbul, Turkey; 3 Department of Bioengineering, Institute of Pure and Applied Sciences, Marmara University, 34722 Istanbul, Turkey; 4 Department of Biochemistry, Faculty of Medicine, Marmara University, 34718 Istanbul, Turkey; alisahin@marmara.edu.tr (A.S.); btlkarademir@gmail.com (B.K.Y.); 5 Genetic and Metabolic Diseases Research and Investigation Center, Marmara University, 34722 Istanbul, Turkey; 6 Department of Medical Microbiology, Marmara University School of Medicine, 34854 Istanbul, Turkey; drbaksu@gmail.com; 7 Metallurgical and Materials Engineering Faculty of Technology, Marmara University, 34722 Istanbul, Turkey; 8 Faculty of Applied Chemistry and Materials Science, University Politehnica of Bucharest, 060042 Bucharest, Romania; denisa.ficai@upb.ro; 9 National Centre for Micro- and Nanomaterials, University Politehnica of Bucharest, 060042 Bucharest, Romania; 10 Industrial Engineering and Management Department, Faculty of Engineering, Lucian Blaga University of Sibiu, 550025 Sibiu, Romania; mihail.titu@ulbsibiu.ro; 11 Academy of Romanian Scientists, 050094 Bucharest, Romania

**Keywords:** corneal patch, electrospinning, microbial keratitis, nanofibers, propolis, *P. aeruginosa*, *S. aureus*

## Abstract

In this research, polyvinyl-alcohol (PVA)/gelatin (GEL)/propolis (Ps) biocompatible nanofiber patches were fabricated via electrospinning technique. The controlled release of Propolis, surface wettability behaviors, antimicrobial activities against the *S. aureus* and *P. aeruginosa*, and biocompatibility properties with the mesenchymal stem cells (MSCs) were investigated in detail. By adding 0.5, 1, and 3 wt.% GEL into the 13 wt.% PVA, the morphological and mechanical results suggested that 13 wt.% PVA/0.5 wt.% GEL patch can be an ideal matrix for 3 and 5 wt.% propolis addition. Morphological results revealed that the diameters of the electrospun nanofiber patches were increased with GEL (from 290 nm to 400 nm) and Ps addition and crosslinking process cause the formation of thicker nanofibers. The tensile strength and elongation at break enhancement were also determined for 13 wt.% PVA/0.5 wt.% GEL/3 wt.% Ps patch. Propolis was released quickly in the first hour and arrived at a plateau. Cell culture and contact angle results confirmed that the 3 wt.% addition of propolis reinforced mesenchymal stem cell proliferation and wettability properties of the patches. The antimicrobial activity demonstrated that propolis loaded patches had antibacterial activity against the *S. aureus*, but for *P. aeruginosa*, more studies should be performed.

## 1. Introduction

The cornea is a protective, transparent, and outer covering of an eyeball. It’s a tissue that acts as a structural barrier and protects the eye against infections, mechanical damage, and ultraviolet (UV) radiation. Two of its main functions are transmission and refraction of incident light beams and contribute to three-fourth of the total refractive power of the eye [1]. The cornea constructs have cellular and acellular elements. The cellular components are composed of epithelial cells, keratocytes, and endothelial cells. The acellular components consist of collagen and glycosaminoglycan. The cornea consists of five layers, the epithelium, Bowman’s layer, stroma, Descemet’s membrane, and endothelium, and, in humans, the central corneal thickness is about 550 μm, and the peripheral thickness is about 620 μm. The thickness is decreasing with age [2]. The cornea diseases are a significant cause of blindness worldwide, being the second, after the cataract, in overall importance. The cornea diseases include infections and inflammation that cause corneal scarring, which ultimately leads to functional blindness [3]. Nowadays, cataract is the cause of approximately 50% of all blindness worldwide, 15% due to trachoma, 10% due to uncorrected refractive error, 4% due to childhood corneal blindness, and 1% due to onchocerciasis. These five diseases are responsible for up to 80% of the world’s blindness [4]. Trachoma is the second leading cause of corneal blindness after the cataract, mainly due to corneal scarring and vascularization. The World Health Organization (WHO) estimates that there are about 146 million people worldwide who have trachoma, and 10 million require surgery to prevent corneal blindness as a result of trachoma. Ocular trauma, corneal ulceration, and childhood corneal blind-ness are other causes of blindness [5]. Corneal ulceration, or microbial keratitis, is a familiar ocular infection that can be caused by fungi, bacteria, viruses, and parasites [6]. *Pseudomonas* species are the most common microbial strains that causes the contact lens-related bacterial ulcers [7] and the post-lens tear surroundings may permit *Pseudomonas* to attach to the corneal epithelium [8,9]. *Staphylococcus aureus (S. aureus)* is another ordinary pathogen of the eye, which has the potential to infect external tissues such as the conjunctiva, tear duct, and cornea. This bacterium allows various toxins and enzymes that can damage tissues and organs, and this damage can cause blindness in the framework of ocular diseases [10]. To treat bacterial damages, it is still not found in precise clinical treatments. Hence, more studies are needed to know the special organism and a therapeutic plan should be formed related to clinical reaction [11].

Electrospinning is one of the best production techniques used in tissue engineering applications to produce tissue scaffolds using various polymers [12]. Electrospinning is used as a method that enables the production of biomimetic scaffolds consisting of a large network of interconnected fibers and pores. The high porosity allows efficient nutrient and metabolic waste exchange between the scaffolds and their surroundings. It also provides a high surface area for the continuous delivery of biochemicals to the seeded cells [13,14,15]. Many different natural and synthetic polymers are widely preferred in many tissue engineering studies, drug delivery systems, and regenerative medicine applications due to their unique properties such as biocompatibility, non-toxicity, and biodegradability [16,17]. PVA in the form of the hydrogel are generally used in tissue engineering, such as arterial phantom, corneal implants, and cartilages [18,19]. It is a hydrophilic, biocompatible, a biodegradable polymer, and it supports the improvement of oxygen permeability [20]. Gelatin is a biocompatible fibrous protein and has moderate antibacterial activity [21]. Its fibrous structure can mimic the collagen fibril structure of the cornea stroma. In Pal et al.’s study, they also fabricated the PVA/Gel film for an artificial skin material [22]. In another study performed by Huang et al., they investigated the comparison of randomly oriented and aligned PVA/Gel nanofibers and reported that cells on PVA-gelatin aligned fibers stretch out extensively [23]. Jain et al. loaded ciprofloxacin in PVA/Gel blends by solution casting method for treatment of corneal ulcers, bacterial keratitis, and other corneal infectious. They proved that PVA/gelatin blends are favorable for controlled release of antibiotic in the eye as compared to the traditional eye drops. [24]. To provide antimicrobial activity against the *S. aureus* and *P. aeruginosa*, propolis was chosen due to its phenolic compounds, which provide antibacterial and antioxidant activity [25,26]. In studies conducted in the literature, with separation techniques such as mass spectroscopy (MS), nuclear magnetic resonance (NMR), chromatography and mass spectroscopy (GC-MS), compounds such as flavonoids, terpenes, phenolics and their esters, sugars, hydrocarbons, and mineral elements have been identified in propolis content [27,28]. The biological activities of propolis are attributed to a variety of major chemical constituents including phenolic acids, phenolic acid esters, flavonoids, and terpenoids such as CAPE, caffeic acid, chrysin, and quercetin, apigenin, kaempferol, pinobanksin 5- ethyl ether, pinocembrin [29]. The main parts of the propolis compounds are flavonoids, which have the 25% ratio [30]. These compounds acquire their antioxidant properties through the lipid peroxidation mechanism [31]. Based on these significant properties, it is widely used in wound healing applications [32].

In this study, PVA, GEL, and propolis which materials are widely used in biomedical applications were used to treatment of corneal keratitis.

## 2. Materials and Method

### 2.1. Materials

Polyvinyl alcohol (PVA, MW = 89,000–98,000) was provided from Sigma Aldrich (Saint Louis, MO, USA), and propolis extract was supplied from SBS (Scientific Bio Solutions) Company, Istanbul, Turkey. Glutaraldehyde (25% solution in water) was provided from Merck KGaA, Darmstadt, Germany.

### 2.2. Fabrication and Characterization of the Electrospinning Solutions

Firstly, 13 wt.% PVA was put into a beaker containing 20 mL of distilled water and dissolved at 300 rpm, 80 °C on magnetic stirrer. After 13% PVA solution dissolved, 0.5, 1, and 3 wt.% GEL were put into this solution. To diminish the surface tension, 3% Tween 80 (Merck KGaA, 64271, Germany) was put into the solutions and stirred for 15 min. After the morphological and mechanical characterizations of 13 wt.% PVA/(0.5, 1, 3)wt.% GEL patches, it was obtained that 13 wt.PVA/0.5 wt.% GEL was better than other concentrations. Therefore, propolis was added directly into 13 wt.% PVA/0.5 wt.% GEL patch to fabricate the propolis added biofunctional patches.

After the preparation of the solutions, these were used to fabricate nanofiber corneal patches via electrospinning. During the electrospinning process; flow rate, voltage, and distance between the collector and needle were optimized. In the electrospinning set-up, a syringe pump (NE-300, New Era Pump Inc., Toledo, OH, USA), a single brass needle (1.63 mm of diameter), and a power supply with high voltage were used with a laboratory-scale electrospinning machine (Inovenso, Istanbul, Turkey). Firstly, polymer solutions were taken into the 10 mL plastic syringes. Then, a high voltage was applied to obtain the Taylor cone. The electrospinning parameters of this study were 24–26 kV, 2–3 mL/h flow rate, 120 mm working distance. As a final stage, 0.25% Glutaraldehyde (GA) was used as a vapor to crosslink the nanofiber patches in the desiccator for 2 h at 40 °C. Then they were dried at room temperature overnight.

### 2.3. Characterization of the Fabricated Corneal Patches

To observe the physicochemical characterizations of the corneal patches, Jasco FT/IR-4700 model machine was performed at room temperature over the range of 4000–400 cm^−1^ in the transmission mode with 4 cm^−1^ resolution (32 scans).

Scanning electron microscopy (SEM, EVO LS 10, ZEISS) was utilized to investigate the morphological structures of the fabricated corneal patches. Before the analysis, patches were coated with gold-palladium for 120 s with a Quorum SC7620 sputter coater. During the analysis, 10 kV accelerating voltage was applied. Image software (Olympus AnalySIS, USA) was employed to measure the average fiber diameter of the SEM images.

To determine the thermal properties of the fabricated corneal patches, differential scanning calorimetry (DSC, Shimadzu, Japan) was employed with a temperature range of 25–300 °C. The heating rate kept constant at 10° C/min for all patches.

In the antimicrobial test, the corneal patches were tested against *S. aureus* and *P. aeruginosa* to observe the antimicrobial activity of the corneal patches. Before the test, *S. aureus* and *P. aeruginosa* were cultured overnight to acquire bacterial suspensions. An automated plate inoculator was utilized to inoculate the bacterial suspensions on Mueller-Hinton agar plates. The fabricated corneal patches were cut to have 5 mm in diameter. The sterilization process was performed with UV light (254 nm) for an hour. As a control group, 2 μg ampicillin was used, and then the disks were cultured at 37 °C for 18 h. After the antimicrobial test finished, the growth inhibition zones were measured.

The uniaxial tensile testing device (Shimadzu Corporation, EZ-LX, Kyoto, Japan) was also used to determine the mechanical behaviors of the patches. Before the measurement, each patch was cut with a 5 cm in length and 1 cm in width mold. The thickness values of each nanofiber patches were measured with a digital micrometer (Mitutoyo, Santa Ana, CA, USA). The test speed was adjusted to 5 mm/min and 5 kN load cell was applied during the test for all patches.

In the drug release test, the first step is the determination of the linear calibration curve. For this purpose, 5 different Ps concentrations were prepared (2, 4, 6, 8, and 10 μg/mL). The drug release analysis was carried out to examine the release behaviors of 3 and 5% Ps into the 13% PVA/0.5% GEL matrix. Firstly, 5 mg of Ps loaded patches were kept in 1 mL PBS (pH 7.4) for 6 h at 37 °C to investigate their release behavior. At predetermined times (0, 0.25, 0.5, 1, 2, 3, 4, and 6 h), 1 mL PBS was taken out from each sample and replaced with 1 mL of fresh PBS. The releasing profile of the Ps was determined at 241 nm by using UV spectroscopy (Shimadzu UV-3600, Kyoto, Japan) and the behaviors were in agreement with the first-order model.

Human adipose-derived mesenchymal stem cells (hADMSCs) were bought from the American Type Culture Collection (ATCC-PCS-500-011). Dulbecco’s Eagle Medium (DMEM) supplemented with 10% fetal bovine serum (FBS), 1% Penicillin/Streptomycin was incubated with cells at 37 °C, in presence of 5% CO_2_ atmosphere. All corneal patches were sterilized with UV in the 24 well plates before the analysis. To observe the cell viability on corneal patches, patches were incubated with DMEM supported with 10% fetal bovine serum, 1% Penicillin/Streptomycin 2 × 10^4^ cells per well. The medium (500 µL) was changed daily. In the MTT protocol; MTT reagent (Sigma) was used to measure the cell viability on the patches, and by using its solution in PBS (5mg/mL) 100 μL was taken from this stock and patches incubated with cells and DMEM for 3 h at 37 °C, 5% CO_2_ for 1, 3, and 7 days. DMEM was removed from the plate, and formazan crystals were dissolved in 500 μL DMSO and detected at 570 nm.

Contact angle measurements were performed to determine the wettability of the corneal patches with the sessile drop method (TGX tensiometer) at room temperature. 3 μL distilled water droplets were dropped on the surface of the nanofiber patches. CCD camera connected to the equipment was used to record the images after 2 s evaluation. The water contact angle values were automatically calculated by the software.

## 3. Results and Discussions

### 3.1. Morphological Properties of the Corneal Patches

**Figure 1:**
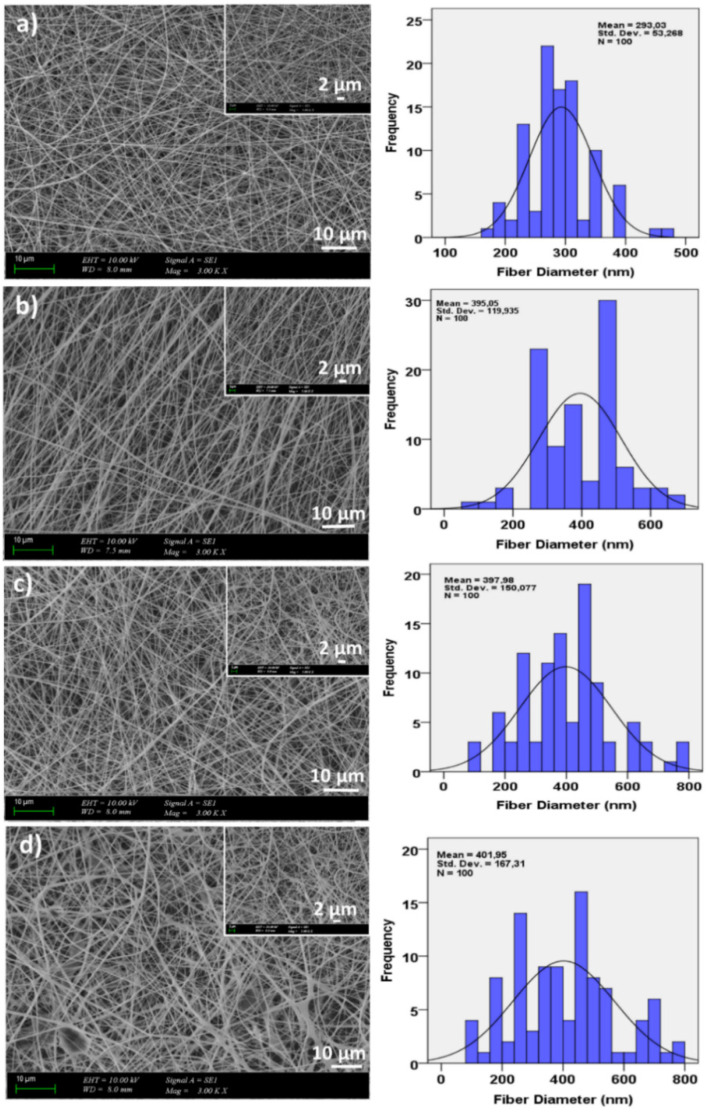
SEM images of the non-crosslinked 13% PVA (**a**), 13% PVA/0.5% GEL (**b**), 13% PVA/1% GEL (**c**), 13% PVA/3% GEL (**d**).

Figure 1 represented the SEM images of the non-crosslinked 13 wt.% PVA and 13 wt.% PVA/(0.5, 1, and 3)wt.% GEL nanofiber patches. The images indicated that all nanofiber patches had homogeneous, continuous, and bead free morphologies. These uniform and smooth morphologies created a porous network to provide diffusion of nutrients and oxygen to the attached cells [33]. The diameters of the electrospun fibers ranged from 293 nm to 401 nm. It was observed that at the constant voltage (26 kV) and flow rate (2 mL/h) values, by the addition of GEL into the 13% PVA, the diameters of the nanofiber patches increased. However, it could be seen that with an increase of GEL concentration, uniform fiber structure without any beads was still preserved.

After adding three different proportions of gel into the PVA polymer, it was observed that the gel preserved the uniform structure of the PVA polymer in all proportions. Still, it was also noted that the nanofiber diameter increased. Additionally, the tensile test showed that by adding GEL into PVA polymer solution, the tensile strength values of the patches decreased. Based on the mechanical and morphological results, 13wt.% PVA/0.5 wt.% GEL was deemed suitable for adding propolis.

**Figure 2:**
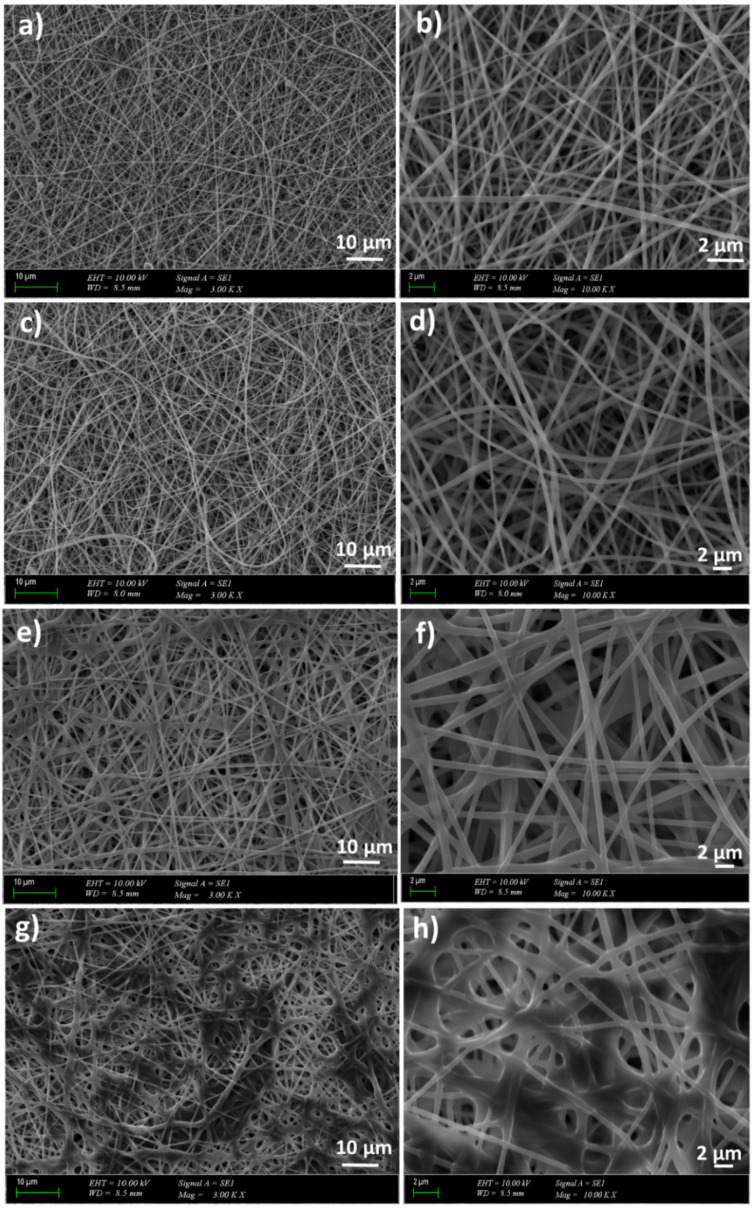
SEM images of the GA-crosslinked 13% PVA (**a**,**b**), 13% PVA/0.5% GEL (**c**,**d**), 13% PVA/0.5% GEL/3% Ps (**e**,**f**), 13% PVA/0.5% GEL/5% Ps (**g**,**h**).

PVA and GEL are water-soluble polymers, so they should be crosslinked for providing water-resistant (stable) biomedical materials [34]. Figure 2 revealed the GA-crosslinked 13 wt.% PVA, 13 wt.% PVA/0.5 wt.% GEL, and 13 wt.% PVA/0.5 wt.% GEL/(3 and 5)wt.% Ps nanofiber patches. It was seen in Figure 2a–d that the crosslinking process did not affect the uniform and beadless structures of the 13 wt.% PVA and 13 wt.% PVA/0.5 wt.% GEL patches. Figure 2e–f showed the smooth and beadless structures of the 13wt.% PVA/0.5 wt.% GEL/3 wt.% Ps nanofiber patches. It can be easily seen that the crosslinking process causes the thicker diameter of the nanofibers. Moreover, the SEM images of the 13 wt.% PVA/0.5 wt.% GEL/5 wt.% Ps also had thicker diameters compared to the non-crosslinked 13 wt.% PVA and 13 wt.% PVA/(0.5, 1, and 3)wt.% GEL nanofiber patches.

### 3.2. FTIR Analysis

**Figure 3:**
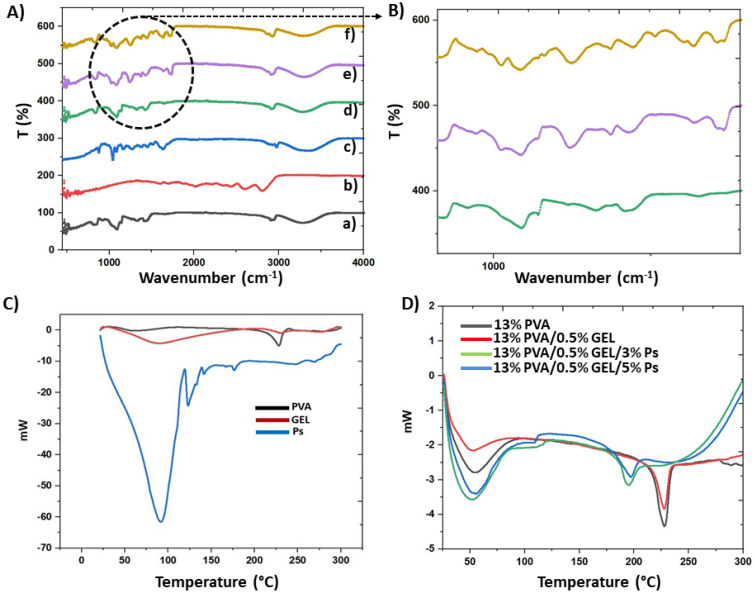
The labels (**A**,**B**) represented the FTIR spectrums: 13% PVA (a), pure GEL (b) and Propolis (c), 13% PVA/0.5% GEL (d), 13% PVA/0.5% GEL/3% Ps (e), 13% PVA/0.5% GEL/5% Ps (f). (**C**,**D**) showed the DSC curves of the pristine PVA, GEL, Ps and prepared corneal patches.

The FTIR spectra of GA crosslinked 13 wt.% PVA, 13 wt.% PVA/(0.5, 1, 3)wt.% GEL, and 13% PVA/(0.5, 1, 3)% GEL/(3 and 5)% Ps patches were shown in Figure 3A,B. In Figure 3A(a), the 13% PVA had main characteristic peaks at ~3268.75 cm^−1^ (O-H group, N-H amino group), ~2910 cm^−1^ (C-H stretch vibration), ~1646.91 cm^−1^, ~1417.42 cm^−1^ (C-O), ~1326.79 cm^−1^ (C-H bending), ~1261.22 cm^−1^ (C=O vibration), ~1085.73 cm^−1^ (C-O group), ~917.95 cm^−1^ (C-C stretching), and ~821.53 cm^−1^ (C-O stretching) [24,25]. Figure 3A(b) represented the FTIR spectrums of the neat GEL which had characteristic peaks at ~3277 cm^−1^, ~2933 cm^−1^, ~1626 cm^−1^, ~1525 cm^−1^, ~1442 cm^−1^, ~1333 cm^−1^, ~1234 cm^−1^, ~1076 cm^−1^, ~1027 cm^−1^, and ~472 cm^−1^. The absorption peaks at 1626 cm^−1^, 1525 cm^−1^, and 1234 cm^−1^ represented the ν_C = O_ and ν_CN_ stretching vibration of groups (amide carbonyl) in Amide I, to δ_NH_ and ν_CN_ vibration of groups in Amide II, and to ν_CN_ and δ_NH_ vibrations in the Amide III band [35]. Propolis extract had main absorption peaks at 3343 cm^−1^, 2973 cm^−1^, 2927 cm^−1^, 1637 cm^−1^, 1513 cm^−1^, 1450 cm^−1^, 1376 cm^−1^, 1270 cm^−1^, 1164 cm^−1^, 1085 cm^−1^, 1043 cm^−1^, and 877 cm^−1^ (Figure 3A(c)) [36]. In Figure 3A(d), the FTIR spectrum of the 13% PVA/0.5% GEL had nearly same spectrum with 13% PVA except the peak 1261 cm^−1^ which observed in FTIR spectrum of the 13% PVA. Additionally, the peak observed at 3270 cm^−1^ shifted to 3278 cm^−1^ with the 0.5% GEL addition. Figure 3A(e) represented the absorbance spectrum of the 13% PVA/0.5% GEL/3% Ps. In this spectrum, propolis addition affected the absorbance spectrum of the 13% PVA/0.5% GEL patch in same points which peaks observed at 1731 cm^−1^, 1637 cm^−1^, 1373 cm^−1^, 1085 cm^−1^, and 1024 cm^−1^. In addition, by adding 5% Ps into the 13% PVA/0.5% GEL the peaks observed at 1710 cm^−1^, 1635 cm^−1^, and 1083 cm^−1^ were due to the PS addition, especially the peak detected at 1513 cm^−1^ (Figure 3A(f)).

### 3.3. Thermal Properties of the Corneal Patches

DSC analysis was performed to assess the thermal behavior of the 13% PVA, 13% PVA/0.5% GEL, and 13% PVA/0.5% GEL/(3 and 5)% Ps nanofiber patches and to examine the miscibility of the blends [34]. Figure 3C,D shown the DSC curves of the pristine PVA, GEL, Ps, and 13% PVA, 13% PVA/0.5% GEL blends at various propolis amounts fabricated by electrospinning. One peak observed at 228 °C in the DSC curve of the pristine PVA and 13% PVA nanofiber patch is attributed to the melting point of PVA [34]. The peak observed at 232.2 °C represented the thermal degradation peak for pristine GEL [37]. Another peak detected at 89.44 °C showed the melting temperature of the pure GEL [38]. When the curve of propolis was examined, two important peaks were observed one is detected at 90.97 °C which represented the water volatilization. Another peak observed at 123.6 °C belonged to the fusion processes of low molecular weight compounds [39]. By adding 0.5% GEL into the 13% PVA, the melting point of the PVA did not change. However, with the addition of 3% Ps and 5% Ps into the 13% PVA/0.5% GEL, the melting point decreased to 198 °C and 196 °C, respectively. A peak in the range of 50–60 °C may be due to the glass transition temperature of the PVA [40]. When 0.5% GEL was added into the 13% PVA, the glass transition point decreases. Moreover, by adding Ps into the 13% PVA/0.5% GEL, the glass transition temperature also reduced again. Miscible blends generally have a single glass transition and melting points in the mixture [41]. The DSC curve obtained in this study also had a single glass transition and melting points, which showed the excellent miscibility of PVA/GEL and PVA/GEL/Ps blends [42].

### 3.4. Antimicrobial Activity of the Fabricated Corneal Patches Against the S. aureus and P. aeruginosa

**Figure 4:**
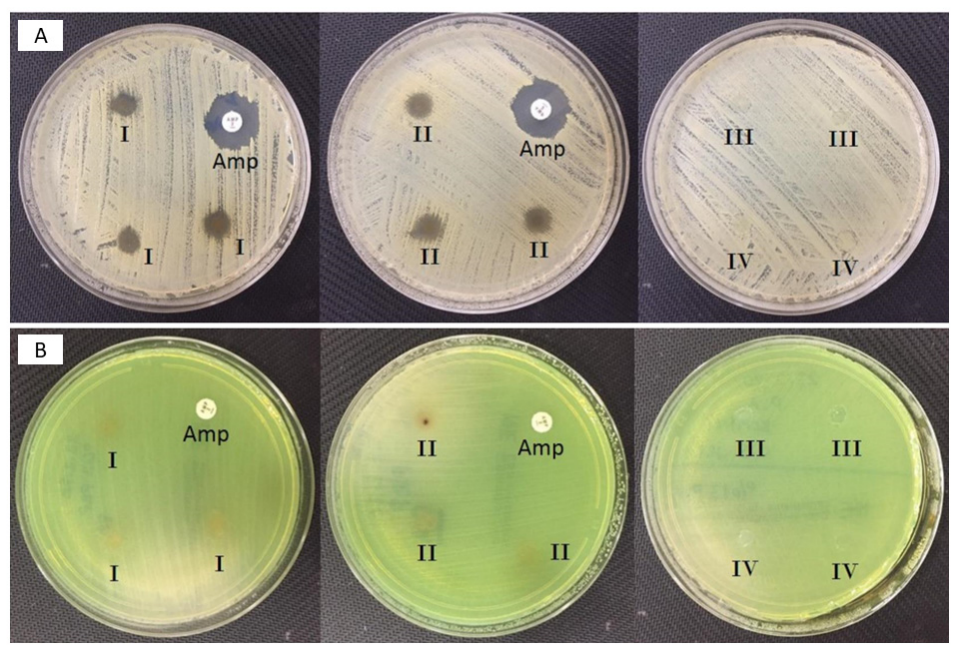
*Staphylococcus aureus* ATCC 29,213 tested by disk diffusion (**A**); I: 3% propolis containing disk, II: 5% propolis containing disk, III: 13% PVA containing disk, IV: 0.5% gel containing disk. *Pseudomonas aeruginosa* ATCC 27,853 tested by disk diffusion (**B**); I: 3% propolis containing disk, II: 5% propolis containing disk, III: 13% PVA containing disk, IV: 0.5% gel containing disk.

Figure 4A showed the antimicrobial activity of both control and propolis-based nanofiber patches against *S. aeurous* and *P. aeruginosa*. The results revealed that propolis added patches had antibacterial activity against the *S. aureus* with 7 mm inhibition zone. The 13% PVA and 13% PVA/0.5% GEL patches were used as a control group in this test. Figure 4B showed the antibacterial activity results of 13% PVA/0.5% GEL/3% Ps and 13% PVA/0.5% GEL/5% Ps nanofiber patches. According to the results, it was observed that propolis extract did not show any antibacterial activity against the *P. aeruginosa*. These results reported that propolis is a good extract for corneal keratitis, but further studies are required. There were performed some studies in the literature about the antibacterial activity of Ps against the *S. aureus*. In Arıkan et al.’s [43] work, propolis added patches showed antibacterial activity against *S. aureus* but did not show antibacterial activity against A. Baumanni and *P. aeruginosa*. In another study, Silici et al. [44] displayed important antibacterial activity against *S. aureus* but did not have antibacterial activity against *E. coli* and *P. aeruginosa*. In Arancı et al.’s [45] work, 3D-printed propolis added alginate scaffolds were fabricated to form wound dressing patches.

### 3.5. Mechanical Properties of the Corneal Patches

**Figure 5:**
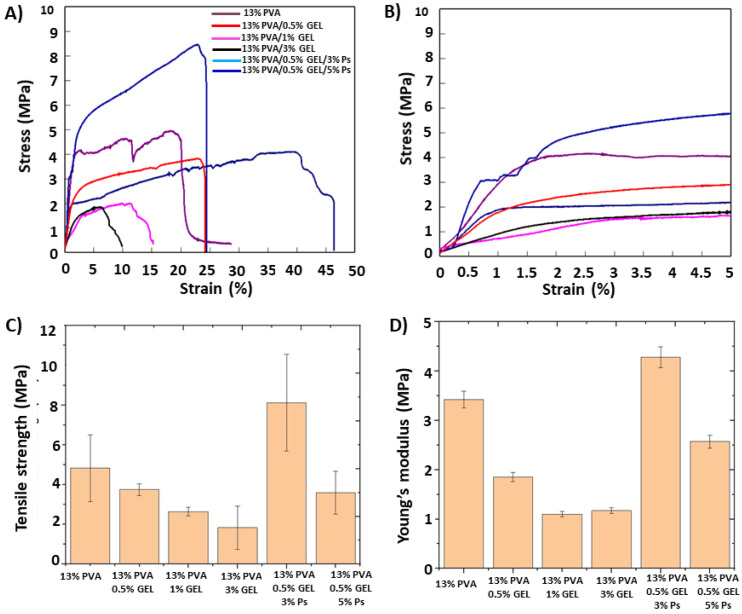
The typical stress/strain curve of the all-nanofiber patches using mean values of three measurements from each other (**A**,**B**), tensile strength (**C**), and calculated Young’s modulus values (**D**) using the linear region of stress strain curve (**B**).

The mechanical strength of ocular transplants is a prominent concern to resist the damage and sustained strength for determining insert performance [24]. The stress-strain behaviors of the electrospun corneal patches were given in Figure 5A–C. The tensile stress values of the 13% PVA/(0.5, 1, and 3)% GEL decreased as the concentration of GEL increased. The elongation at break percentage of the 13% PVA increased with 0.5% GEL addition from 13.86% to 36.32%. However, by adding 1% and 3% GEL into the 13% PVA matrix, the elongation at break percentages decreased sharply. Therefore, the amount of 0.5% GEL was determined as the ratio to add propolis, and it was obtained that with the addition of 3% Ps into the 13% PVA/0.5% GEL, the tensile stress values increased again from 3.75 MPa to 8.12 MPa, propolis acting as a reinforcing agent. The adhesive qualities of propolis can be useful to increase the tensile stress values of PVA/GEL fibers [46]. On the other hand, elongation at break value (27.69%) was lower than the value of 13% PVA/0.5% GEL (36 and 32%), but still higher than other GEL concentrations. By adding 5% Ps into the 13% PVA/0.5% GEL, the tensile stress and elongation at break values decreased again. This can be explained due to the existence of propolis particles, which can prevent the precise orientation of polymer molecules and along with the heterogeneous structure cause a decrease in tensile strength values. If all patches are compared between each other, it can be said that 13% PVA, 13% PVA/0.5% GEL, and 13% PVA/0.5% GEL/3% Ps had acceptable strength values for cornea tissue regeneration (3–5 MPa) [47]. The Young modulus of the nanofiber patches was calculated using the linear region of the stress/strain curve (Figure 5B). The results were revealed in Figure 5D with a column graph. According to the results, the same trend was observed between the patches like the tensile strength values of the patches. By adding 0.5, 1, and 3% GEL into the 13% PVA, the elastic modulus values of the patches decreased slightly. However, with the addition of 3% Ps into the 13% PVA/0.5% GEL, the elastic modulus of the nanofiber patches increased again from 1.85 MPa to 4.28 MPa. The elastic modulus of the 13% PVA/0.5% GEL/5% Ps was found as 2.57 MPa and proved that 5% Ps ratio was too high to form mechanically corneal strength patches.

### 3.6. Drug Release Profiles of Propolis

**Figure 6:**
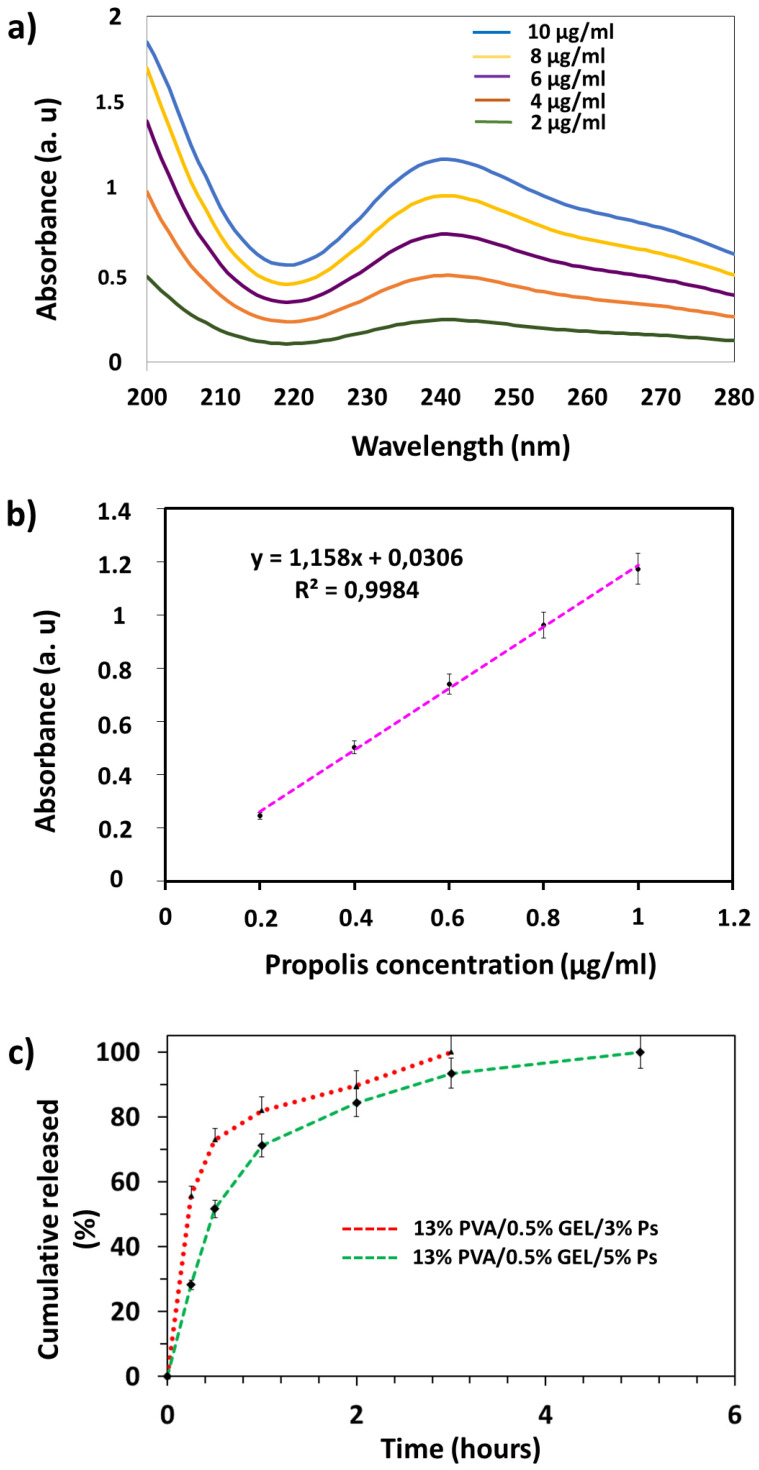
In vitro drug release profiles of scaffolds: Ps calibration curve (**a**), Absorption spectra of Ps at different amounts (**b**), Ps cumulative release graph (**c**).

In vitro, drug release analyzes of propolis-loaded nanofiber patches were performed. Firstly, for the quantitative determination of drug release (Figure 6a), a linear standard calibration curve was constructed from propolis absorption values (R^2^ = 0.9984) obtained and UV spectra (Figure 6b) obtained in the concentration range of propolis from 2 to 10 μg/mL. Propolis released was detected by UV 241 nm absorbance. In order to mimic the physiological conditions of living organisms, the release profiles of propolis loaded nanofibers were analyzed at 37 °C and pH 7.4 in PBS. In vitro release studies were performed for 6 h to evaluate the release kinetics of propolis loaded nanofibers. As shown in Figure 6c, although the release rates were different at the two various propolis concentrations, both propolis loaded nanofibers showed burst drug release within the first 1 h. This was primarily attributed to the high water solubility of PVA and Gel. Propolis release rates reached 82% and 71.14% in the first 1 h for 3% and 5%, respectively. The drug release of nanofibers loaded with 3% propolis reached 100% at the end of approximately 3 h, while the release of approximately the entire nanofiber loaded with 5% propolis occurred at the end of the 5th h. According to the result obtained here, it is seen that the length of time of drug release is directly proportional to the amount of propolis loaded. Propolis release analysis from PVA hydrogels was performed in the study conducted by Oliveira et al. In the release analysis that lasted for 4 days in total, and it was reported that propolis exhibited a burst release profile on the 1st day and no prolonged release was observed. If the propolis concentration increased from 8% to 52%, the release time increased as the amount of propolis increased [25]. In different research in the literature, the rapid release profile of propolis was observed at the beginning of the experiment. However, the in vitro release was made more controlled by increasing the propolis concentration [34]. The data we obtained in this study proved similar to the studies in the literature that propolis demonstrates a rapid release in water-soluble polymers and the release time increases according to the increasing amount.

### 3.7. Biocompatibility Properties of the Corneal Nanofiber Patches

**Figure 7:**
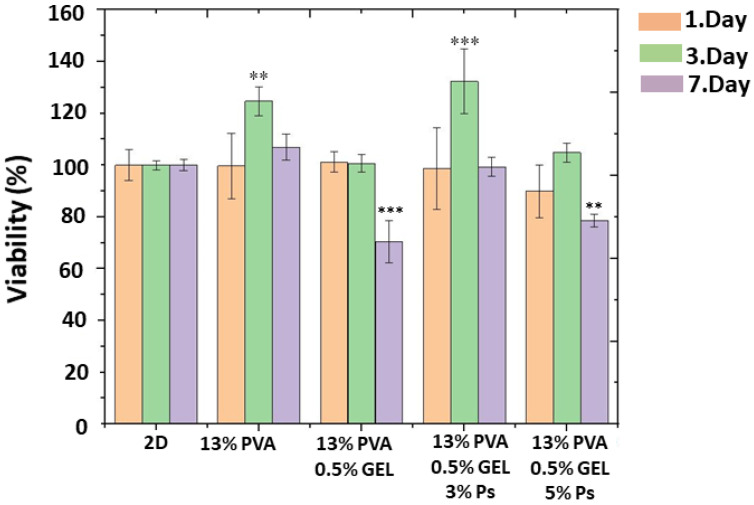
MTT result of the fabricated corneal nanofiber patches and 2D (MSCs) after 1, 3, and 7 days of incubation. The mark “**” points the significant difference *p* < 0.01 and the mark “***” represents the significant difference *p* < 0.001.

The MTT assay test, which is a rapid and sensitive technique, is the initial step to evaluate the biological properties of the obtained structures and to examine cell viability and cell proliferation [48]. The cytocompatibility of MSCs on the PVA/GEL and propolis added PVA/GEL patches were shown in Figure 7. According to the cytotoxicity results for the first-day incubation, acceptable viability values of the cells were detected on the 13% PVA (99%), 13% PVA/0.5% GEL (101%), 13% PVA/0.5% GEL/3% Ps (99%), and 13% PVA/0.5% GEL/5% Ps (89%). On the 2nd day, the viability of the cells on the patches had more than 100% percentages which demonstrated that cells were proliferated on the patches more than the first day. On the other hand, the viability of the cells decreased on the 7th day of incubation, but they still had acceptable values. Moreover, the viability of the cells on the 13% PVA still higher than the control group (2D cells). When the samples containing propolis were compared among themselves, it was observed that the cell viability rate in the patch containing 3% propolis was higher for all incubation times and this can be also correlated with the higher concentration of propolis. This situation can be due to less porous structures and higher diameter values related to the thick fibers [49]. However, we conclude that both 3% and 5% propolis added patches are suitable for producing biocompatible corneal patches (to see the biological performance of the patches visually, the fluorescence and SEM images of the MSCs on the patches are reported in the Supplementary Materials Figure S1).

### 3.8. Surface Wettability Properties of the Corneal Nanofiber Patches

**Figure 8:**
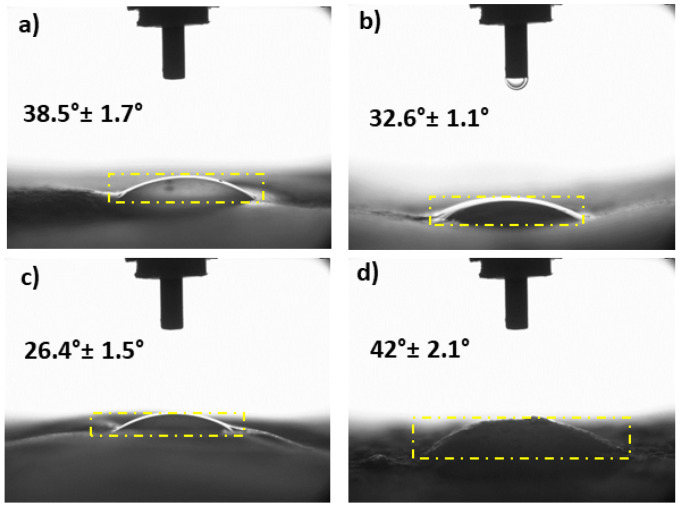
Water contact angle properties of the 13% PVA (**a**), 13% PVA/0.5% GEL (**b**), 13% PVA/0.5% GEL/3% Ps (**c**), 13% PVA/0.5% GEL/5% Ps (**d**).

The hydrophobic (contact angle > 90) and hydrophilic (contact angle < 90°) possessions of the samples have a critical role in the interactions between the extracellular matrix and cells [50,51]. Figure 8 showed the surface wettability properties of the corneal nanofiber patches with their contact angles. The contact angle of the 13% PVA was 38.5° ± 1.7° approving the hydrophilicity of the patch. The blend 13% PVA/0.5% GEL demonstrated lower contact angle (32.6° ± 1.1°) compared to the 13% PVA. This might be due to multiple surface variations such as phase separation, the hydrophilicity of gel, roughness changes, etc. [52]. By adding 3% Ps into the 13% PVA/0.5% GEL, the contact angle become 26.4° ± 1.5° indicating that propolis addition decreased the contact angle of the PVA/GEL blend. This proved that the surface of the 13% PVA/0.5% GEL/3% Ps patch was the most hydrophilic compared to the other patches indicating that droplets diffused in more on the surface [53]. On the other hand, 5% Ps ratio increased the contact angle to 42° ± 2.1°, which showed that a higher amount of propolis decreased the hydrophilicity of the 13% PVA/0.5% GEL proving the hydrophobic nature of the propolis [54,55].

## 4. Conclusions

In this study, electrospun biocompatible PVA/GEL/Ps nanofiber patches were fabricated to provide the antimicrobial activity against the *S. aureus* and *P. aeruginosa,* which are the common microorganisms cause the corneal keratitis. The fabricated corneal nanofiber patches were crosslinked with Glutaraldehyde to provide long stability-to avoid the rapid solubilization. Propolis has biocompatibility, and it is a bioactive substance which properties are essential for functional tissue production. According to the SEM results, it can conclude that patches fabricated with electrospinning method are in the nanometer scale, and results demonstrated that the crosslinking process improve the stability (reduce the fast solubilization) and did not alter the morphology of the patches negatively. Antimicrobial test results reported that propolis-based patches showed antibacterial activity against the *S. aureus.* The 13% PVA/0.5% GEL/3% Ps patch has a high potential due to its proper mechanical properties, notable hydrophilicity, and cell attachment. In the release process, the burst release of propolis was detected in the first hour followed by a sustained release. Based on this study, we reported, for the first time, the functionality and potential of propolis-based patches for the treatment of the corneal keratitis using mesenchymal stem cells, and we demonstrated successful production of corneal patches at a nanometer scale to mimic the structure of the innate cornea tissue.

## Supplementary Materials

The following are available online: Figure S1: Fluorescence images of the DAPI stained 13% PVA (a), 13% PVA/0.5% GEL (c), 13% PVA/0.5% GEL/3% Ps (e), 13% PVA/0.5% GEL/5% Ps (g) nanofiber patches, Cell morphology on the 13% PVA (b), 13% PVA/0.5% GEL (d), 13% PVA/0.5% GEL/3% Ps (f), 13% PVA/0.5% GEL/5% Ps (i).

## Sample Availability

Samples of the patches are available from the authors.
