# Supplementary material for: Propolis-Based Nanofiber Patches to Repair Corneal Microbial Keratitis
Source: Molecules. 2021 Apr 28;26(9):2577. doi: 10.3390/molecules26092577 (PMC8125036; doi:10.3390/molecules26092577)
Supplement: Supplementary file 1 [file molecules-26-02577-s001.zip › molecules-1176819-supplementary.pdf]

Supplementary Materials for

Propolis-Based Nanofiber Patches to Repair Corneal Microbial Keratitis

Songul Ulag [1,2], Elif Ilhan [1,3], Ramazan Demirhan [1], Ali Sahin [4,5], Betul Karademir Yilmaz [4,5], Burak Aksu [6],
Mustafa Sengor [1,7], Denisa Ficai [8,9], Aurel Mihail Titu [10,11], Anton Ficai [8,9,11,*] and Oguzhan Gunduz [1,7,*]

1. Center for Nanotechnology & Biomaterials Application and Research (NBUAM), Marmara University, 34722 İstanbul, Turkey; ulagitu1773@gmail.com (S.U.); eliffguven@gmail.com (E.I.); r.demirhan@outlook.com (R.D.); mustafasengor@gmail.com (M.S.)
2. Metallurgical and Materials Engineering, Institute of Pure and Applied Sciences, Marmara University, 34722 İstanbul, Turkey
3. Department of Bioengineering, Institute of Pure and Applied Sciences, Marmara University, 2 34722 Istanbul, Turkey
4. Department of Biochemistry, Faculty of Medicine, Marmara University, 34718 İstanbul, Turkey; alisahin@marmara.edu.tr (A.S.); btlkarademir@gmail.com (B.K.Y.)
5. Genetic and Metabolic Diseases Research and Investigation Center, Marmara University, Istanbul, Turkey
6. Department of Medical Microbiology, Marmara University School of Medicine, 34854 Istanbul, Turkey; drbaksu@gmail.com
7. Metallurgical and Materials Engineering Faculty of Technology, Marmara University, 34722 Istanbul, Turkey
8. Faculty of Applied Chemistry and Materials Science, University Politehnica of Bucharest, 060042 Bucharest, Romania; denisa.ficai@upb.ro
9. National Centre for Micro- and Nanomaterials, University Politehnica of Bucharest, 060042 Bucharest, Romania
10. Industrial Engineering and Management Department, Faculty of Engineering, Lucian Blaga University of Sibiu, 550025 Sibiu, Romania; mihail.titu@ulbsibiu.ro
11. Academy of Romanian Scientists, 050094 Bucharest, Romania;

* Correspondence: anton.ficai@upb.ro (A.F.); ucemogu@ucl.ac.uk (O.G.)

Protocols for fixation and DAPI staining

Fixation protocol was performed to observe the cell morphology on the corneal patches. Firstly, the growth medium was removed from the plate, and all patches were fixed with 4% glutaraldehyde. And then patches were dehydrated with diluted ethanol. After that, they were dried at room temperature. Before the SEM analysis, dried patches were coated with Au for 60 s at 10 kV.
To investigate the attachment of MSCs on the corneal patches, DAPI staining was carried out. Firstly, the growth medium was taken away from the plates, and they were rinsed with PBS. Then, patches were fixed with 4% formaldehyde for half an hour at room temperature. After that, they were rinsed with PBS. After that 1 μg/mL DAPI (Invitrogen) was put on each patch to stain the nucleus of the cells within a time interval 20 min at room temperature. Finally, DAPI solutions were taken, and patches were observed the images under an inverted fluorescence microscope (Leica).

Biocompatibility properties of the corneal nanofiber patches

To observe the nanofiber-cell interactions of 13% PVA, 13% PVA/0.5% GEL, and Ps added patches, SEM micrographs of MSCs on patches were taken after 7 days of cell culture. Figure S1 (b, d, f, and h) demonstrated the cell growth is higher on 13% PVA/0.5% GEL/(3 and 5)% Ps patches than on 13% PVA

and 13% PVA/0.5% GEL patches. Morphology of cells on patches revealed that the MSCs were well spread and attached on the surface of the propolis added nanofiber patches than pristine PVA and PVA/GEL nanofibers. The reason for better attachment of MSCs on the propolis-based patches may be due to better hydrophilicity of the propolis added patches[47].

Fluorescence images in Figure S1 (a, c, e, and g) were detected to observe the attachment of MSCs over the nanofiber patches after 1 day of incubation. Cell attachment and spreading over the patches or scaffolds could be considered as an important stage for cell growth which is an essential property for the regeneration of the damaged tissues[51]. As seen in the images (Figure S1 (a, c, e, and g)), the cells showed nearly the same spindle-like morphology for 13% PVA, 13% PVA/0.5% GEL, and 13% PVA/0.5% GEL/3% Ps patches and the number of cells on these patches were higher than 13% PVA/0.5% GEL/5% Ps patch. This situation is similar to MTT results. As a result of MTT, the viability values for 13% PVA/0.5% GEL/5% Ps patch were lower than the patches containing 3%Ps and other patches without propolis for all incubation times. However, in Bilginer *et al.* [52] work, they fabricated scaffolds with 5, 10, 15, and 20 % propolis amounts with electrospinning technique. They found that cells spread and attach on scaffolds by the increasing of propolis content. Based on the SEM images in Figure 2 (g, h), it can be said that the morphology of the 13% PVA/0.5% GEL/5% Ps seemed to more affected by the crosslinking process and had a thicker diameter than other patches. Therefore, this situation may be having a negative effect on the viability of the cells. Since the morphology of eukaryotic cells is closely related to their function, observations of cell cultures confirm the cytotoxic and anti-proliferative effect the high amount of propolis on stem cells. Reported studies of the effect on cell viability included high or mild cytotoxicity, and even pro-proliferative activity of this product, depending on the concentration, exposure time and in vitro/in vivo conditions [1].

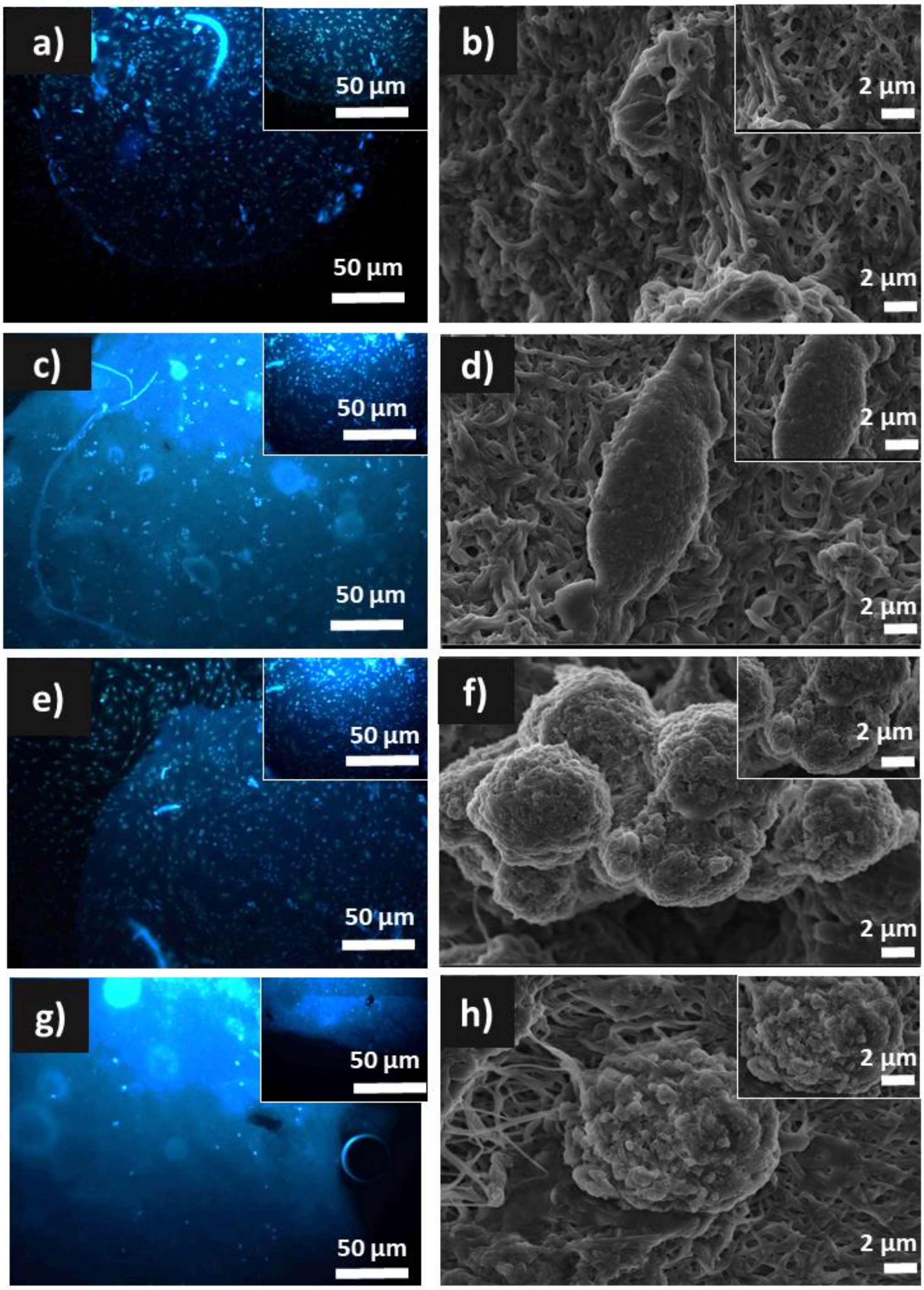

Figure S1. Fluorescence images of the DAPI stained 13% PVA (a), 13% PVA/0.5% GEL (c), 13% PVA/0.5% GEL/3% Ps (e), 13% PVA/0.5% GEL/5% Ps (g) nanofiber patches, Cell morphology on the 13% PVA (b), 13% PVA/0.5% GEL (d), 13% PVA/0.5% GEL/3% Ps (f), 13% PVA/0.5% GEL/5% Ps (i).

References

1. Tyszka-Czochara, M.; Pasko, P.; Reczyński, W.; Szlósarczyk, M.; Bystrowska.; Opoka, W. Zinc and Propolis Reduces Cytotoxicity and Proliferation in Skin Fibroblast Cell Culture: Total Polyphenol Content and Antioxidant Capacity of Propolis. *Biol Trace Elem Res.* **2014**, 160, 123–131, doi: 10.1007/s12011-014-0019-3.
